# PAICS contributes to gastric carcinogenesis and participates in DNA damage response by interacting with histone deacetylase 1/2

**DOI:** 10.1038/s41419-020-2708-5

**Published:** 2020-07-06

**Authors:** Nan Huang, Chang Xu, Liang Deng, Xue Li, Zhixuan Bian, Yue Zhang, Shuping Long, Yan Chen, Ni Zhen, Guohui Li, Fenyong Sun

**Affiliations:** 1 Department of Clinical Laboratory Medicine, Shanghai Tenth People’s Hospital of Tongji University, Shanghai, China; 2 Institute of Life Sciences, Jiangsu University, Zhenjiang, China; 3 Department of Laboratory Medicine, Shanghai Children’s Medical Center, Shanghai Jiao Tong University School of Medicine, Shanghai, China; 4 Department of Central Laboratory, Shanghai Tenth People’s Hospital of Tongji University, Shanghai, 200072 China

**Keywords:** Gastric cancer, Apoptosis, Target identification, DNA damage response, Double-strand DNA breaks

## Abstract

Phosphoribosylaminoimidazole carboxylase, phosphoribosylaminoimidazole succinocarboxamide synthetase (PAICS), an essential enzyme involved in de novo purine biosynthesis, is connected with formation of various tumors. However, the specific biological roles and related mechanisms of PAICS in gastric cancer (GC) remain unclear. In the present study, we identified for the first time that PAICS was significantly upregulated in GC and high expression of PAICS was correlated with poor prognosis of patients with GC. In addition, knockdown of PAICS significantly induced cell apoptosis, and inhibited GC cell growth both in vitro and in vivo. Mechanistic studies first found that PAICS was engaged in DNA damage response, and knockdown of PAICS in GC cell lines induced DNA damage and impaired DNA damage repair efficiency. Further explorations revealed that PAICS interacted with histone deacetylase HDAC1 and HDAC2, and PAICS deficiency decreased the expression of DAD51 and inhibited its recruitment to DNA damage sites by impairing HDAC1/2 deacetylase activity, eventually preventing DNA damage repair. Consistently, PAICS deficiency enhanced the sensitivity of GC cells to DNA damage agent, cisplatin (CDDP), both in vitro and in vivo. Altogether, our findings demonstrate that PAICS plays an oncogenic role in GC, which act as a novel diagnosis and prognostic biomarker for patients with GC.

## Introduction

Gastric cancer (GC) is the fifth most common malignancy and third leading cause of cancer-related death worldwide with the annual death rate in China accounting for more than 40% of the world^1^. Except for early surgical resection, chemotherapy is the main treatment for GC, which effectively prolongs survival and improves related symptoms of patients in stage IV^2,3^. However, the prognosis of most patients with advanced GC remains poor mostly because of chemotherapy resistance, which is typically caused by the highly heterogeneous and numerous genetic alterations of this malignancy^4^. Therefore, numerous studies should been conducted to explore the precise molecular mechanisms underlying the initiation and progression of GC to develop more effective interventions.

Phosphoribosylaminoimidazole carboxylase, phosphoribosylaminoimidazole succinocarboxamide synthetase (PAICS) is a putative bifunctional enzyme involved in the de novo purine biosynthesis pathway with both 5-aminoimidazole ribonucleotide carboxylase and 4-(N-succinylcarbox-amide)-5-aminoimidazole ribonucleotide synthetase activities^5^. Previous study has demonstrated that cancer cells rely on the PAICS-dependent metabolic pathway for adenosine monophosphate and guanosine monophosphate synthesis, and inactivation of the de novo pathway inhibits cancer cell proliferation both in vitro and in vivo^6^. PAICS has been shown to be highly expressed in various types of cancer such as breast cancer^7^, prostate cancer^8^, bladder cancer^9^, lung cancer^6,10^, and acute lymphoblastic leukemia^11^. Moreover, overexpression of PAICS is predictive of the poor survival of patients with tumors^6,8–10,12^. A study of the oncogenic mechanism of PAICS has revealed that PAICS can induce epithelial-mesenchymal transition in bladder cancer by positively regulating SNAI1 and reducing E-cadherin expression^9^. In addition, the loss of MYC occupancy was observed on the PAICS promoter in presence of JQ1 in prostate cancer^8^. However, the specific biological roles and related mechanisms of PAICS in GC remain unclear.

The DNA damage response (DDR) greatly impacts genomic stability through DNA repair pathways, which ultimately determine cell fate^13^. Normal cells use precisely regulated DDR processes to protect their DNA from exogenous and endogenous DNA-damaging factors^14,15^. DNA double-strand breaks (DSBs) are the most serious type of DNA damage, as they result in the loss of chromosomal regions. The non-homologous DNA end joining (NHEJ) pathway and homologous recombination repair (HR) pathway are predominantly responsible for the restoration of DSBs^16^. Upon DDR, DNA damage sites are identified by the MRE11-DAD51-NBS1 (MRN) complex, which then recruits ataxia telangiectasia mutated kinase to these sites^17–19^. This kinase is activated immediately by phosphorylation, which then phosphorylates downstream effectors such as H2AX^20^. Mediator of DNA damage checkpoint protein 1 then binds to the phosphorylated H2AX (γH2AX) as a platform for DNA repair^18,21,22^. NHEJ repair mainly occurs in G1-phase through the recruitment of effectors such as 53BP1, Ku70/Ku80 complexes, which can promote the downstream proteins such as XRCC to repair the damaged DNA^23^. HR repair mainly occurs in S-G2M phase and involves the recruitment of effectors such as BRCA1/2 and DAD51, which can use sister chromatid as template for precise repair^24–26^. When DNA repair processes are dysfunctional, genetic predisposition to cancer tends to arise^27^.

In the present study, we investigated the role of PAICS in GC and explored related mechanisms of action. We found that PAICS expression was exceptionally upregulated in GC tissues, and high expression of PAICS was correlated with poor prognosis of patients with GC. In addition, PAICS inhibited GC cell apoptosis and promoted GC cell proliferation both in vitro and in vivo. More importantly, PAICS was engaged in DDR by directly interacting with HDAC1/2 and affected DNA damage repair by targeting DAD51. Significantly, knockdown of PAICS enhanced the sensitivity of GC cells to cisplatin (CDDP) both in vitro and in vivo, which provides new insights into GC therapy.

## Results

### PAICS is upregulated in GC tissues and related to the prognosis of patients with GC

**Fig. 1:**
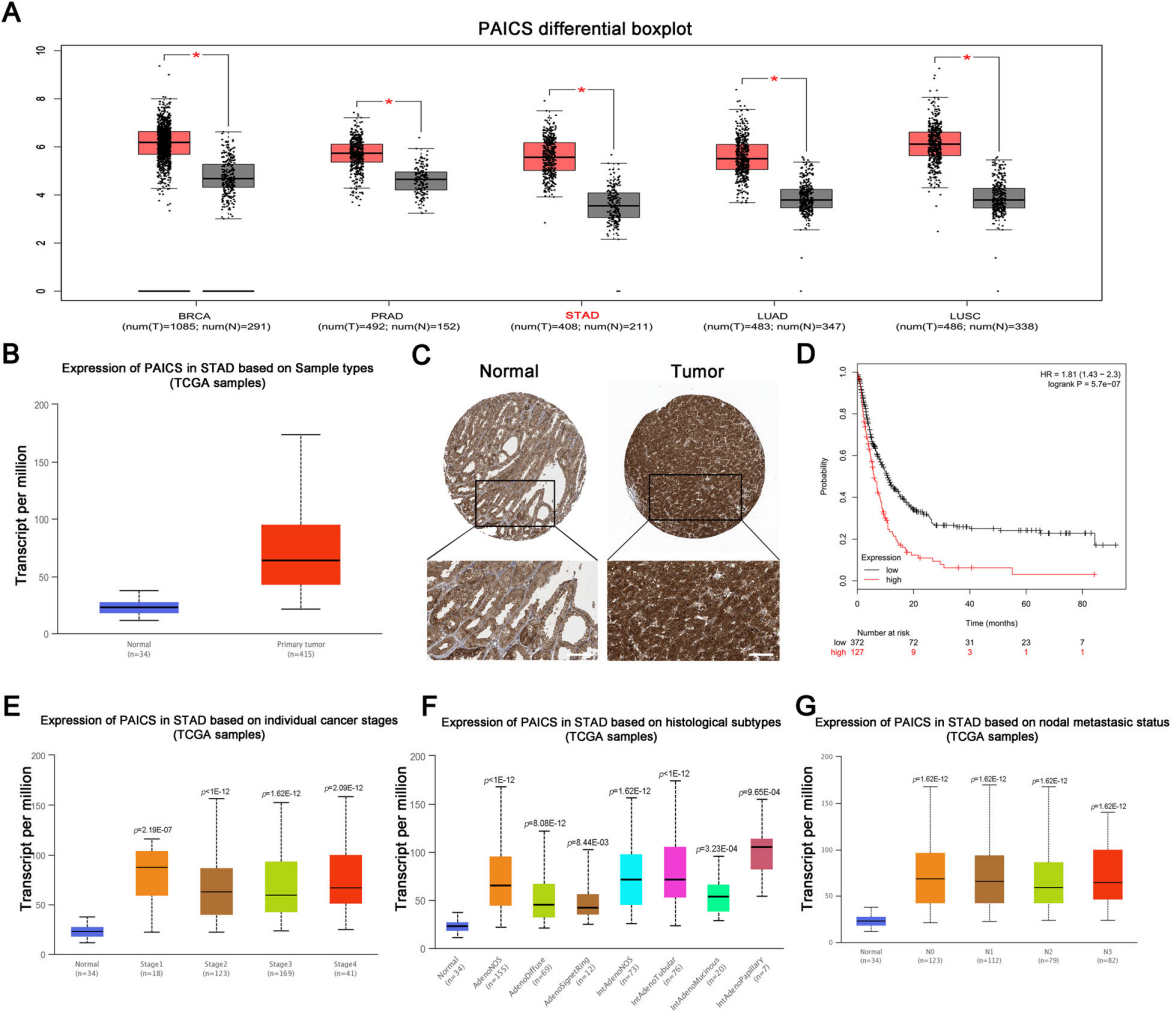
PAICS is upregulated and serves as a prognostic factor in GC. **a** Boxplots represent PAICS expression level in breast invasive carcinoma (BRCA), prostate adenocarcinoma (PRAD), stomach adenocarcinoma (STAD), lung adenocarcinoma (LUAD), lung squamous cell carcinoma (LUSC) and paired non-tumor tissues. **b** Relative expression of PAICS mRNA in normal and primary GC samples from TCGA. **c** Immunohistochemical analysis of PAICS in normal gastric tissue and GC tissue provided by the Human Protein Atlas project. Scale bar 50 μm. **d** Kaplan–Meier survival curves of overall survival in 499 GC patients based on PAICS expression level. **e** Relative expression of PAICS mRNA transcript in normal and stage 1, 2, 3 and 4 GC patients. **f** Relative expression of PAICS mRNA transcript in different histological subtypes. **g** Relative expression of PAICS mRNA transcript in normal and stage N0, N1, N2, and N3 GC patients.

We first analyzed the expression of PAICS using the GEPIA database. The results indicated that PAICS was significantly overexpressed in various cancer tissues, including GC (Fig. 1a). We then confirmed the upregulated expression of PAICS in GC using the ULCAN database which contains 449 GC samples (*p* < 1 × 10^−12^) (Fig. 1b). In addition, we examined the immunohistochemistry staining results from the Human Protein Atlas project and found that PAICS was strongly expressed in GC tissues but weakly expressed in normal tissues (Fig. 1c). The Kaplan–Meier survival curves showed that GC patients with high PAICS expression had a poorer prognosis and worse overall survival rates compared to those with low PAICS expression (*p* = 5.7 × 10^−7^) (Fig. 1d). However, subsequent analysis revealed that PAICS expression was not correlated with cancer stage, subtype, or TNM stage (Fig. 1e–g).

### Knockdown of PAICS inhibits GC cell proliferation and promotes cell apoptosis in vitro

**Fig. 2:**
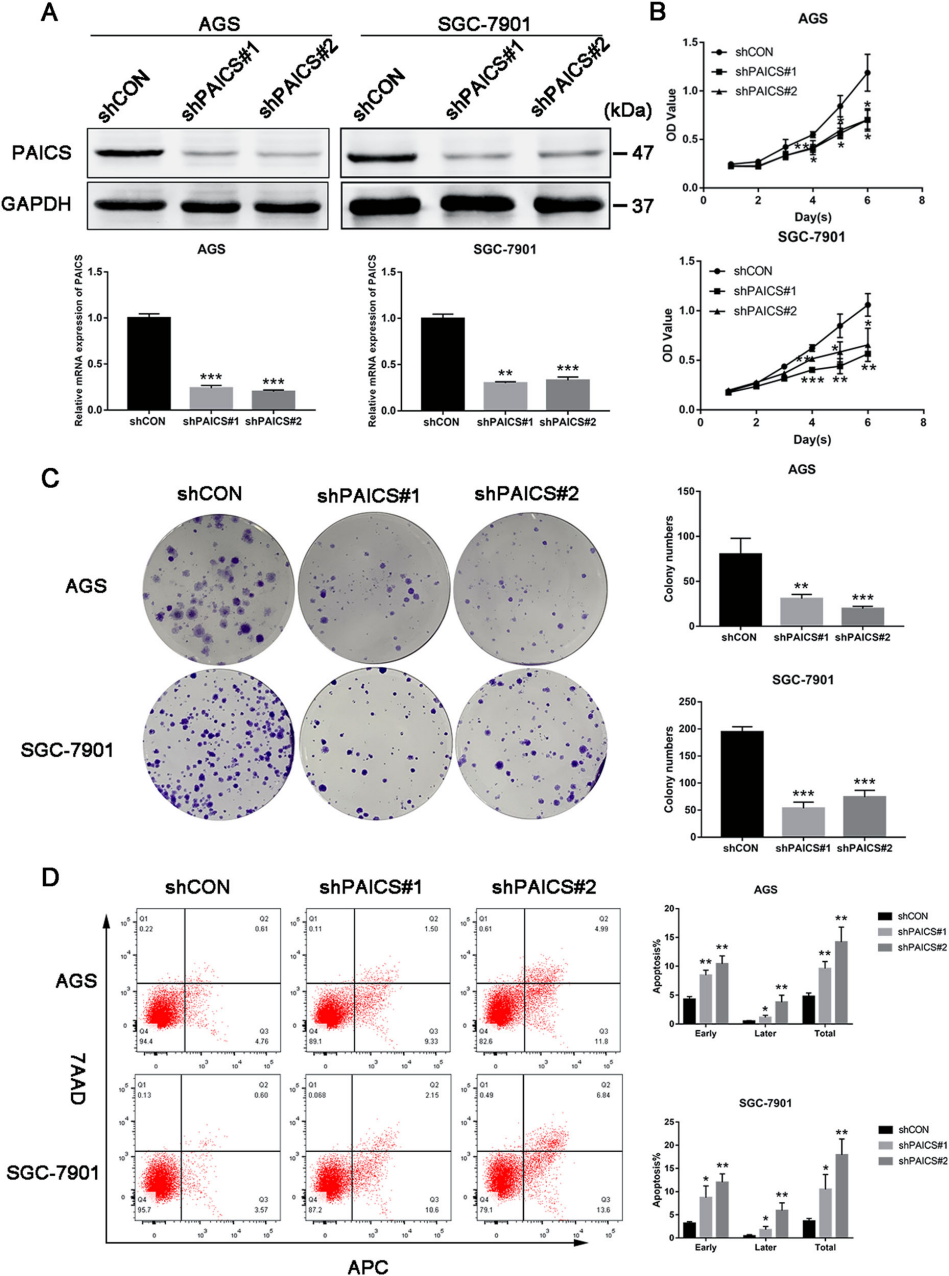
PAICS promotes GC cell proliferation and inhibits apoptosis in vitro. **a** Western blot and qRT-PCR showed PAICS-knockdown efficiency in AGS and SGC-7901 cells. Cell proliferation (**b**), colony formation (**c**), and apoptosis (**d**) of GC cell lines (AGS and SGC-7901) with or without PAICS knockdown. All data are shown as mean ± SD from three independent experiments. ***p < 0.001, **p < 0.01, *p < 0.05.

We further investigated the role of PAICS in GC cell proliferation and apoptosis in vitro. Considering the high PAICS expression in GC, PAICS expression in the GC (AGS and SGC-7901) cell lines was therefore downregulated using short hairpin RNA-encoding lentiviruses (shCON, shPAICS#1, or shPAICS#2). Results of qRT-PCR and western blotting confirmed the consistent knockdown of PAICS in both the AGS and SGC-7901 cell lines (Fig. 2a). Data from CCK8 assay indicated that cell proliferation was suppressed with knockdown of PAICS (Fig. 2b). Consistently, knockdown of PAICS significantly decreased cell colony formation rate (Fig. 2c). In addition, cell apoptosis analysis by flow cytometry demonstrated an increased percentage of apoptosis in PAICS-knockdown cells (Fig. 2d). This finding was further supported by increases in cleaved caspases 3/8/9 proteins and a decrease in antiapoptotic protein Bcl-2 in the shPAICS groups compared with the shCON group (Supplementary Fig. S1a). Meanwhile, knockdown of PAICS significantly increased the activity of caspase-3 (Supplementary Fig. S1b). We also performed cell-cycle analysis and observed a significant decrease in the G1-phase and an increase in the S-phase, which suggested that PAICS knockdown disrupted cell-cycle progression with S-phase arrest (Supplementary Fig. S1c). Western blots showed that PAICS knockdown significantly decreased levels of S-phase-related proteins including CDC25A, cyclin A2 and CDK2 (Supplementary Fig. S1d). We further overexpressed PAICS in the stable knockdown cell line SGC-7901 (Supplementary Fig. S2a) and performed rescue assays to confirm the effects of PAICS on cell proliferation. Data from CCK8 assay showed that PAICS overexpression rescued the growth-inhibitory effect of GC cells induced by PAICS knockdown (Supplementary Fig. S2b). Moreover, we knockeddown expression of PAICS in the normal gastric epithelial cell line GES-1 and performed CCK8 assay to detect cell proliferation. Our results showed that knockdown of PAICS in normal gastric cells had little effect on cell proliferation (Supplementary Fig. S3a).

### Knockdown of PAICS inhibits gastric carcinogenesis in vivo

**Fig. 3:**
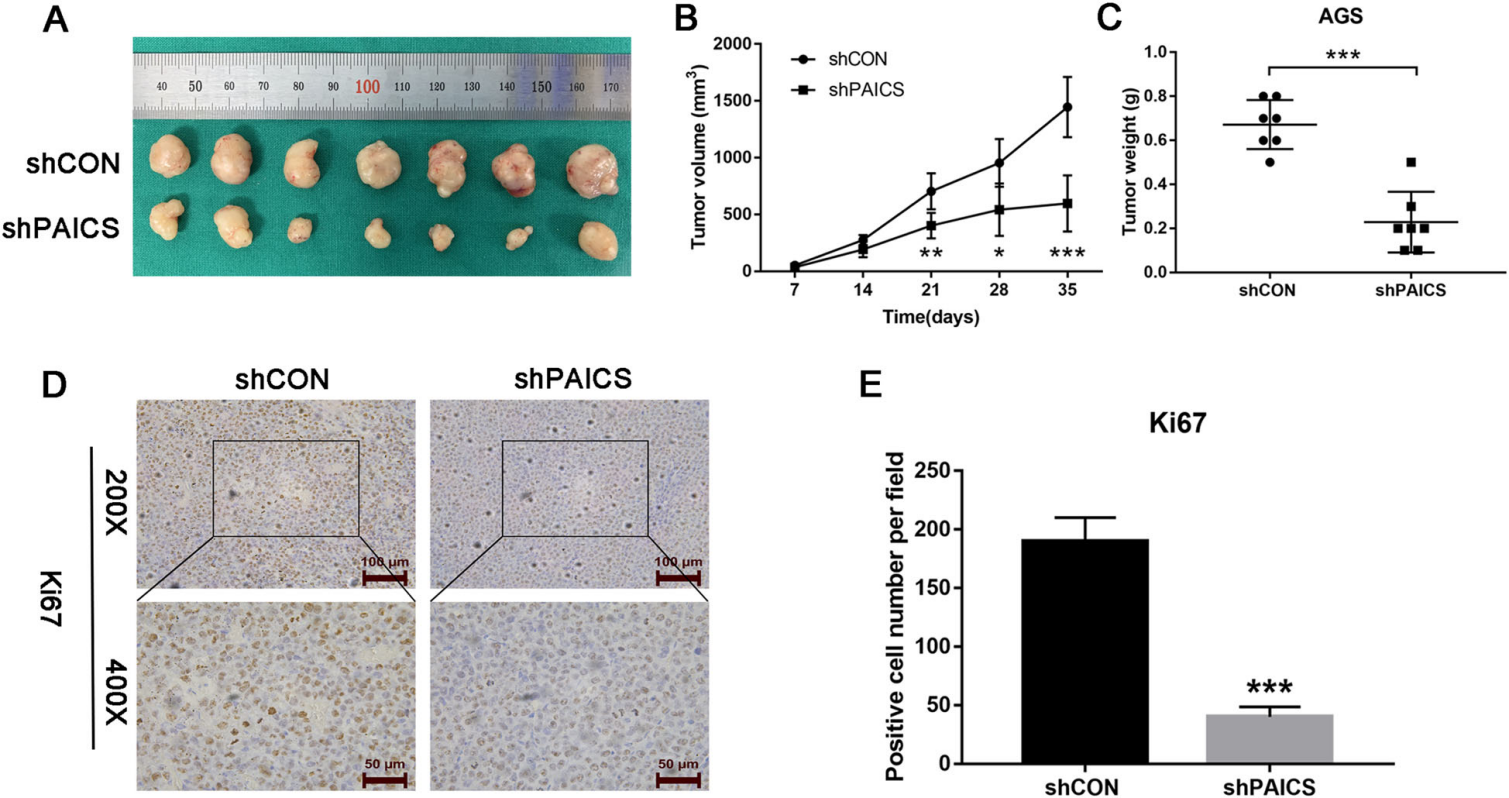
PAICS promotes gastric carcinogenesis in vivo. **a** Subcutaneous tumor model of shCON- or shPAICS-AGS cells. **b**, **c** Tumor volume and weight were measured at the indicated weeks after mice were transplanted. **d**, **e** IHC analysis of Ki67 expression levels in tumor tissues obtained from the xenograft mice. The histogram indicates the Ki67 positive cells from panel. Data represent the mean ± SD from three independent experiments, ***p < 0.001, **p < 0.01, *p < 0.05.

The effect of PAICS on tumor growth in vivo was further evaluated by establishing a subcutaneous xenograft tumor model in nude mice using PAICS-knockdown or the control AGS cells. As expected, tumor growth was dramatically slower in the PAICS-knockdown group (shPAICS) compared with the control group (shCON) (Fig. 3a, b). In addition, similar results were observed in the mean tumor weight (Fig. 3c). Consistently, we also observed a significant decrease in the positive rate of Ki67 in the PAICS-knockdown group by immunohistochemistry analysis (Fig. 3d, e). In addition, we performed flow cytometry to measure the Ki67 level in PAICS-knockdown (shPAICS#1, shPAICS#2) and the corresponding control (shCON) cells and found that knockdown of PAICS decreased the percentage of Ki67 positive cells (Supplementary Fig. S3b).

### PAICS is engaged in DDR in GC cells

**Fig. 4:**
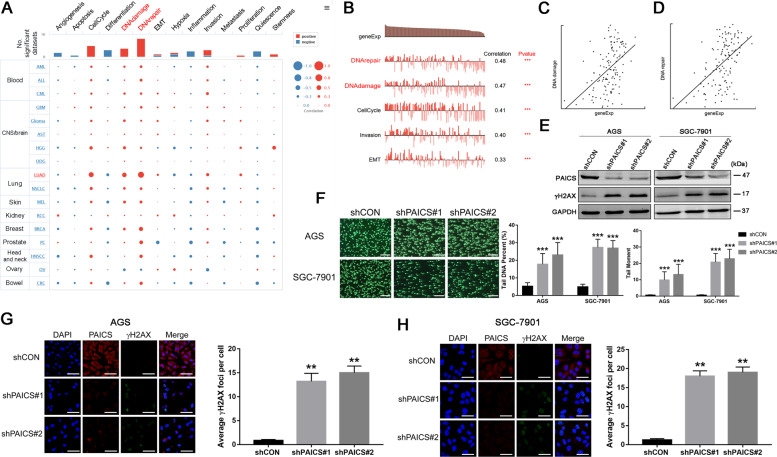
PAICS is engaged in DNA damage repair in GC cells. **a** Single-cell analysis indicates that PAICS is primarily involved in regulation of the DNA damage and repair pathway. **b**–**d** Data from Kim KT (No. of cells = 126) demonstrate that PAICS mRNA expression is positively correlated with regulation of the DNA damage (**c**) and repair (**d**). **e** Western blot analysis of expression of γH2AX protein in shCON- or shPAICS-GC cells. **f** Representative images and quantification of comet assay of shCON- or shPAICS-GC cells. Scale bar 500 μm. **g**, **h** Immunofluorescence analysis of the formation of γH2AX foci in shCON- or shPAICS-GC cells. Scale bar 50 μm. Data represent the mean ± SD from three independent experiments, ***p < 0.001, **p < 0.01, *p < 0.05.

To explore the functions of PAICS, CancerSEA was first used for single-cell analysis. The results indicated that PAICS was primarily involved in regulating the DNA damage and repair (Fig. 4a–d). To confirm this results, we first performed western blotting to investigate the expression levels of the DNA damage-related protein γH2AX in PAICS-knockdown or the control GC cell lines and found that knockdown of PAICS prominently increased γH2AX expression (Fig. 4e). In addition, the neutral comet assay showed that knockdown of PAICS significantly increased the tail DNA in GC cell lines, suggesting that PAICS deletion sustained the DNA damage (Fig. 4f). Concurrently, immunofluorescence analysis confirmed a marked increase in γH2AX foci formation with the knockdown of PAICS (Fig. 4g, h). We also performed rescue assays to confirm the effects of PAICS on DNA damage. Results of the neutral comet assay showed that PAICS knockdown induced DNA damage, which was indicated by a significant increase in tail DNA of GC cells; PAICS overexpression rescued the phenotype of GC cells induced by PAICS knockdown (Supplementary Fig. S2c).

### PAICS is involved in DDR by interacting with HDAC1 and HDAC2

**Fig. 5:**
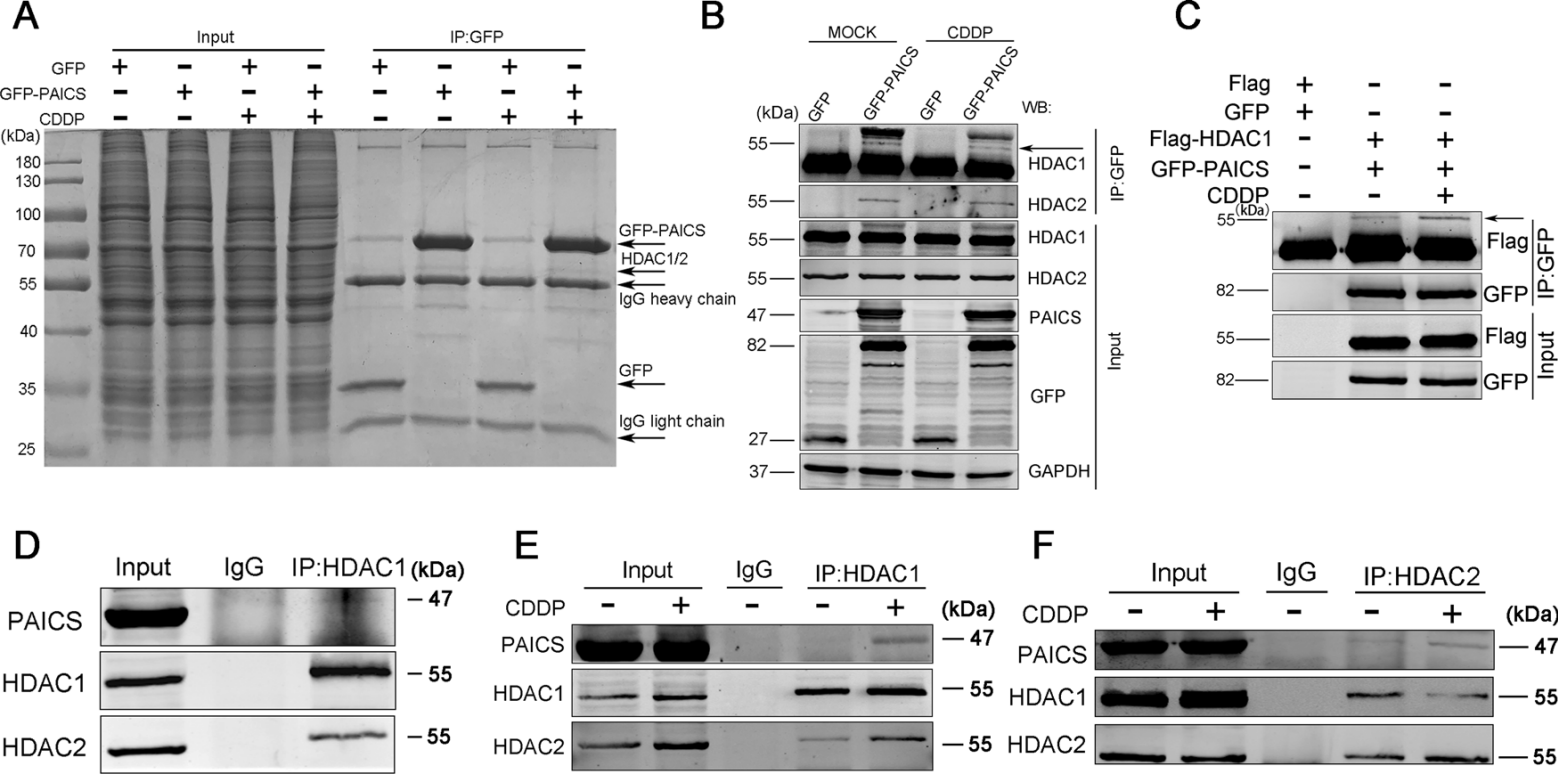
PAICS interacts with HDAC1 and HDAC2. **a**, **b** HEK293T cells transfected with GFP-vector or GFP-PAICS were stimulated with or without CDDP (10 µg/mL for 12 h followed by a 2-h recovery). Cell lysates were immunoprecipitated using anti-GFP. Immunoprecipitants or the whole-cell lysates (Input) separated by SDS-PAGE gel were stained with coomassie brilliant blue for mass spectrometric analysis (**a**); immunoprecipitants or the Input were analyzed by western blot with anti-HDAC1 and anti-HDAC2 (**b**). **c** HEK293T cells co-transfected with Flag-vector/GFP-vector or Flag-HDAC1/GFP-PAICS were stimulated with or without CDDP (10 µg/mL for 12 h followed by a 2-h recovery). Cell lysates were immunoprecipitated using anti-GFP and immunoprecipitants or the Input were analyzed by western blot with anti-GFP and anti-Flag. **d** SGC-7901 cells lysates were immunoprecipitated using anti-HDAC1 and immunoprecipitants or the Input were analyzed by western blot with anti-PAICS, anti-HDAC1 or anti-HDAC2. **e**, **f** SGC-7901 cells stimulated with or without CDDP (10 µg/mL for 12 h followed by a 2-h recovery) were collected. Cell lysates were immunoprecipitated using anti-HDAC1 or anti-HDAC2 and immunoprecipitants or the Input were analyzed by western blot with anti-PAICS, anti-HDAC1 or anti-HDAC2.

To further explore the detailed mechanism of PAICS involved in DDR, co-immunoprecipitation and mass spectrometry assays were performed to identity the potential binding proteins of PAICS. The results suggested that PAICS interacted with HDAC1 (Fig. 5a and Table S1). Considering that HDAC1 often forms a complex with HDAC2 to participate in DDR, we therefore evaluated the interaction of PAICS with both HDAC1 and HDAC2 using co-immunoprecipitation assay. We transfected HEK293T cells with GFP-vector or GFP-tagged PAICS in the presence or absence of CDDP treatment and observed that PAICS interacted with both HDAC1 and HDAC2 (Fig. 5b). Concurrently, the interaction was confirmed by co-transfecting Flag-tagged HDAC1 with GFP-tagged PAICS in HEK293T cells (Fig. 5c). We also confirmed the interaction of PAICS and HDAC1/2 in SGC-7901 cells via endogenous immunocoprecipitation (Fig. 5d). In addition, we observed a much stronger interaction of PAICS with HDAC1/2 in response to CDDP treatment (Fig. 5e, f), suggesting that the HDAC-PAICS complex was involved in the DDR.

### PAICS deficiency decreases DAD51 expression and inhibited its recruitment to DNA damage sites

**Fig. 6:**
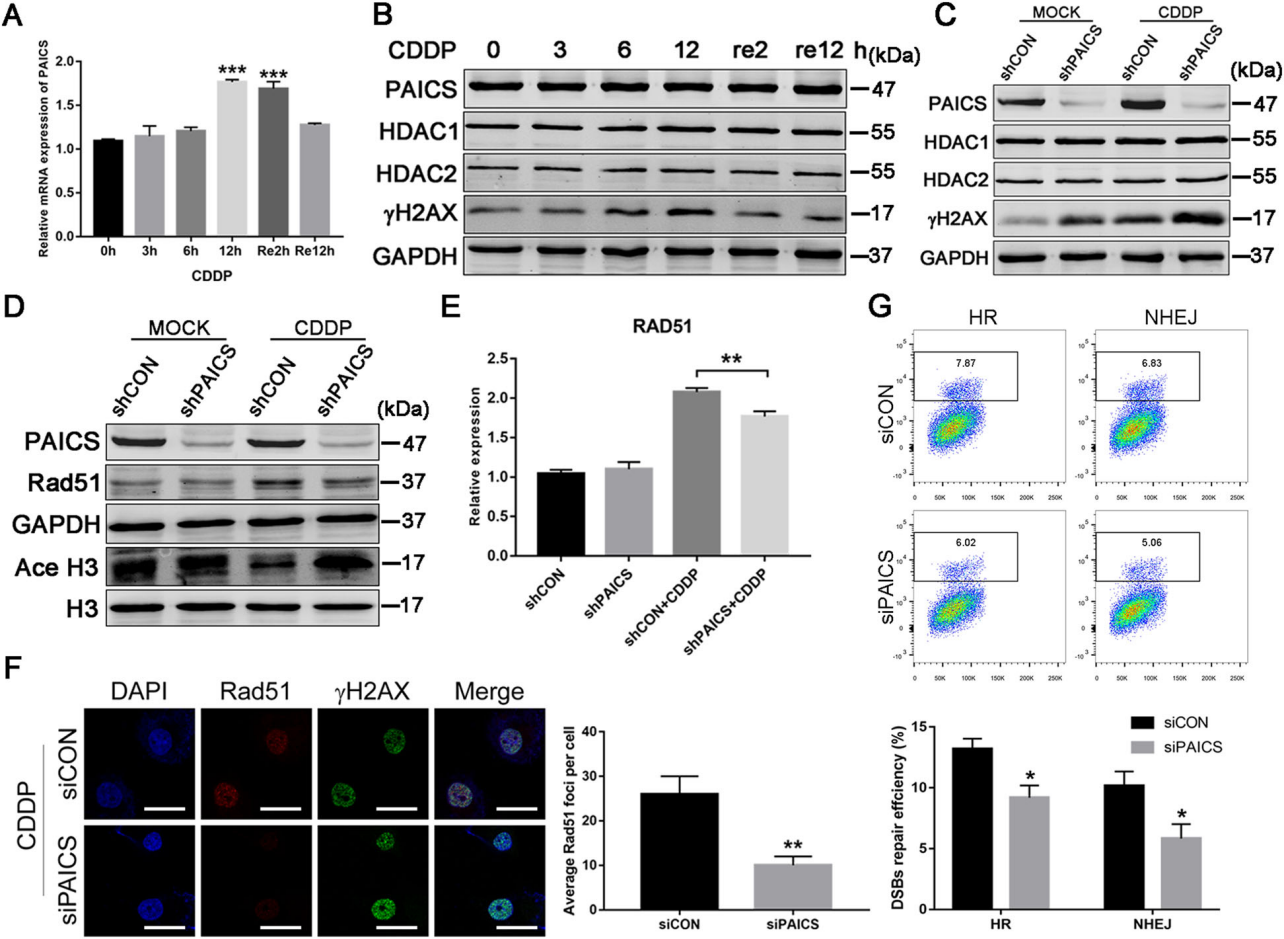
Knockdown of PAICS decreases DAD51 expression and inhibited its recruitment to DNA damage sites. **a** qRT-PCR analysis of PAICS mRNA expression in SGC-7901 cells treated with CDDP (10 µg/mL for 3, 6, or 12 h followed by 2- or 12-h recovery). **b** Western blot analysis of PAICS, HDAC1/2 proteins levels in SGC-7901 cells treated with CDDP (10 µg/mL for 3, 6, or 12 h followed by 2- or 12-h recovery). **c** Western blot analysis of HDAC1 or HDAC2 protein levels in shCON- or shPAICS-SGC-7901 cells stimulated with or without CDDP (10 µg/mL for 12 h followed by a 2 h recovery). **d** Western blot analysis of acetylated-H3K56 (Ace H3), DAD51 proteins levels in shCON- or shPAICS-SGC-7901 cells stimulated with or without CDDP (10 µg/mL for 12 h followed by a 2 h recovery). **e** qRT-PCR analysis of DAD51 mRNA expression in shCON- or shPAICS-SGC-7901 cells stimulated with or without CDDP (10 µg/mL for 12 h followed by a 2-h recovery). **f** Immunofluorescence analysis of the formation of DAD51 foci in shCON- or shPAICS-SGC-7901 cells stimulated with CDDP (10 µg/mL for 12 h followed by a 2-h recovery). Scale bar 50 μm. **g** Flow cytometry detection of HR and NHEJ repair efficiency in 293 T cells transiently transfected with siPAICS or siCON for 48 h. Data represent the mean ± SD from three independent experiments, ***p < 0.001, **p < 0.01, *p < 0.05.

We further detected the mRNA levels of PAICS in SGC-7901 cells treated with CDDP at different time points to explore the detailed regulatory mechanism of HDAC-PAICS in the DDR. The results showed increased PAICS mRNA levels in response to the DDR induced by CDDP as well as a decrease in the level of this gene with recovery (Fig. 6a). We also evaluated the PAICS protein levels. Interestingly, we found no obvious changes in PAICS protein expression either in CDDP-induced DDR or its recovery (Fig. 6b). Moreover, no obvious changes in HDAC1 or HDAC2 protein levels were observed (Fig. 6b). We next determined whether PAICS could regulate HDAC1/2 protein expression in the presence or absence of CDDP treatment and found no obvious effects on the expression of HDAC1 or HDAC2 protein (Fig. 6c). Upon DNA damage, HDAC1/2 is rapidly recruited to DNA damage sites to participate in DNA damage repair by promoting histone H3 lysine 56 (H3K56) deacetylation^28,29^. To further explore whether PAICS could affect the deacetylase activity of HDAC1/2, we then determined the acetylation level of H3K56 in the presence or absence of CDDP treatment and found a decrease in acetylated-H3K56 in CDDP-induced DDR, which was blocked by PAICS knockdown (Fig. 6d). This finding suggested that knockdown of PAICS affected the deacetylase activity of HDAC1/2. To confirm the effect of PAICS on HDAC1/2 deacetylation activity, we performed HDAC activity assay and found that knockdown of PAICS significantly decreased HDAC deacetylation activity in the presence of CDDP treatment (Supplementary Fig. S4). Notably, our results showed that knockdown of PAICS had no obvious effect on HDAC deacetylation activity in the absence of CDDP treatment (Supplementary Fig. S4). Previous studies have demonstrated that inhibiting the deacetylase activity of HDAC1/2 can reduce the expression and recruitment of repair-related proteins such as DAD51 to DNA damage sites, thus affecting the efficiency of DNA damage repair^30–33^. We then evaluated the expression of DAD51 and found that knockdown of PAICS significantly decreased the levels of DAD51 protein and mRNA in the presence of CDDP treatment (Fig. 6d, e). Concurrently, results of immunofluorescence assay showed that PAICS deficiency reduced the recruitment of DAD51 to DNA damage sites in the presence of CDDP-induced DDR (Fig. 6f). In addition, we performed the HR and NHEJ assays and found that knockdown of PAICS inhibited both HR and NHEJ repair pathways (Fig. 6g).

### PAICS deficiency enhances the sensitivity of GC cells to CDDP both in vitro and in vivo

**Fig. 7:**
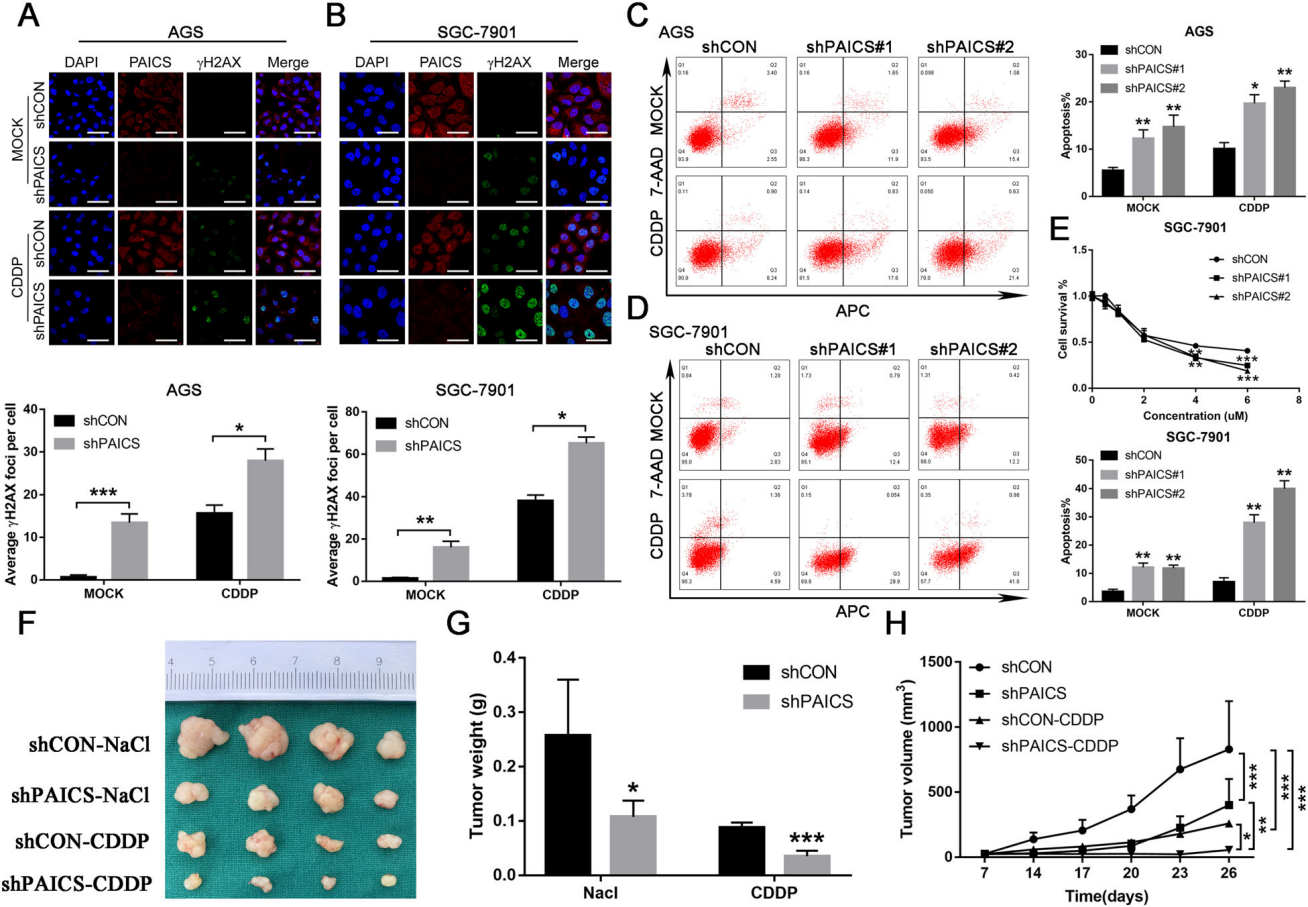
PAICS deficiency enhances the sensitivity of GC cells to CDDP both in vitro and in vivo. **a**, **b** Immunofluorescence analysis of the formation of γH2AX foci in shCON- or shPAICS-GC cells stimulated with or without CDDP (10 µg/mL for 12 h followed by a 2-h recovery). Scale bar 50 μm. **c**, **d** Flow cytometry detection of cell apoptosis in shCON- or shPAICS-GC cells (AGS and SGC-7901) treated with or without CDDP (10 µg/mL for 12 h followed by a 2-h recovery). **e** CCK-8 detection of cell viability in shCON- or shPAICS-SGC-7901 cells treated with increasing doses of CDDP for 48 h. **f** Subcutaneous tumor model of shCON- or shPAICS-SGC-7901 treated with CDDP or saline solution (0.9% NaCl). **g**, **h** Tumor volume and weight were measured at the indicated days after mice were transplanted. All data are shown as mean ± SD from three independent experiments. ***p < 0.001, **p < 0.01, *p < 0.05.

Impaired DNA damage repair induced by HDAC inhibition can sensitize tumor cells to DNA-damaging agents^34–36^. We therefore investigated whether knockdown of PAICS could enhance the sensitivity of GC cells to CDDP, a well-known DNA-damaging agent for GC. Results of immunofluorescence showed that knockdown of PAICS in both AGS and SGC-7901 cell lines significantly increased the formation of γH2AX foci induced by CDDP treatment (Fig. 7a, b), suggesting that PAICS deletion enhanced the sensitivity of GC cells to CDDP. Flow cytometry evaluation confirmed that PAICS knockdown markedly sensitized GC cell lines to apoptosis induction by CDDP (Fig. 7c, d). We also observed that knockdown of PAICS significantly enhanced the inhibition of cell viability in SGC-7901 cells induced by CDDP treatment (Fig. 7e). Moreover, knockdown of PAICS significantly enhanced the growth inhibition of GC cells induced by CDDP treatment in the subcutaneous xenograft tumor model in vivo (Fig. 7f–h).

## Discussion

**Fig. 8:**
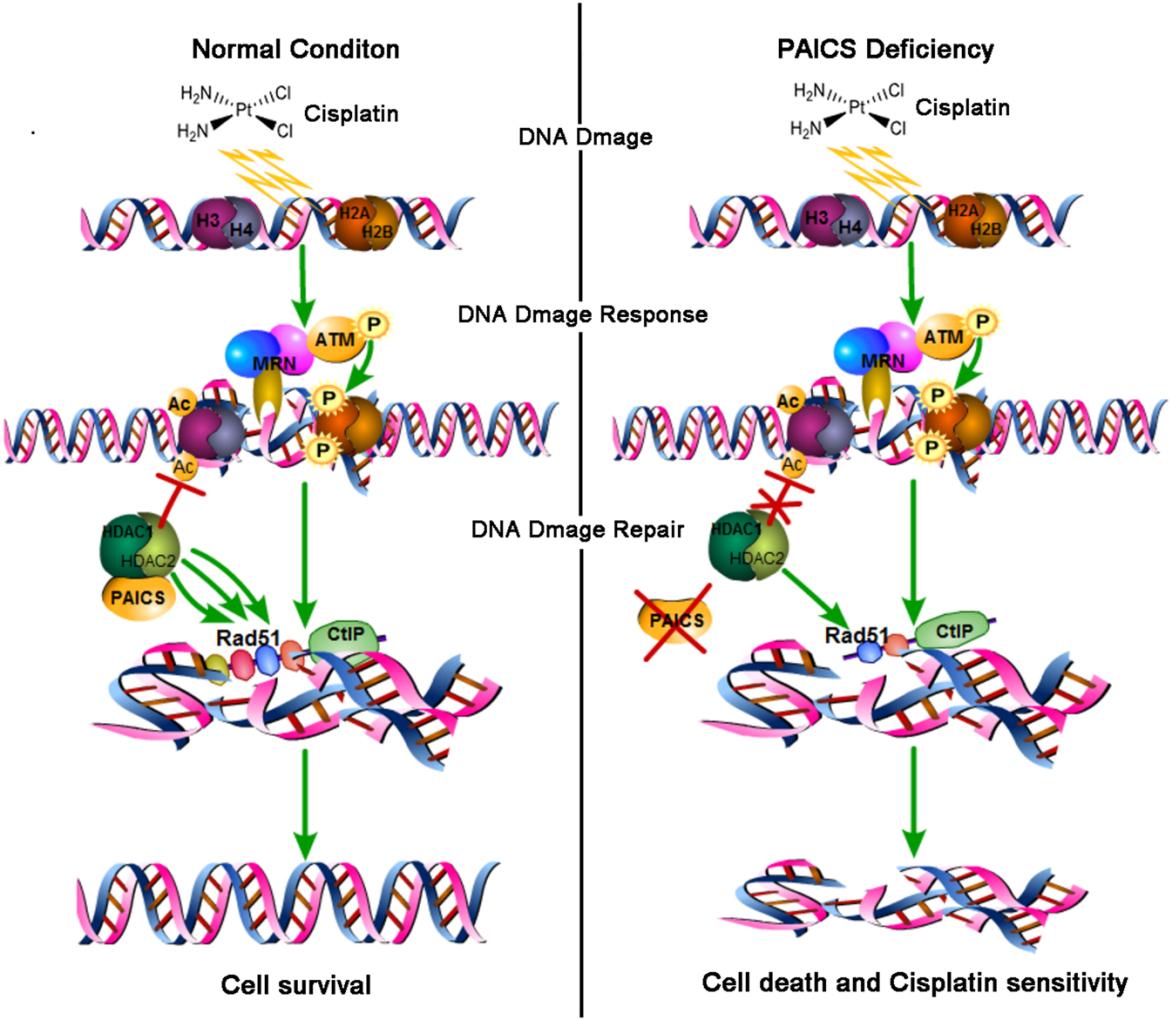
Under normal conditions, PAICS is involved in DNA damage response, and cells can repair the DNA damage induced by cisplatin and survive (Left); Under PAICS-deficient conditions, the DNA damage induced by cisplatin could not be repaired, which increases cell death and cisplatin sensitivity (Right). Proposed mechanism for PAICS-induced gastric carcinogenesis and cisplatin sensitivity.

As a key regulatory enzyme involved in the *de novo* purine biosynthesis pathway, PAICS plays an important role in maintaining normal metabolism^5,6^. Recent studies have demonstrated elevated expression of PAICS in various malignancies^6,8–10,12^, suggesting that PAICS affects the initiation and progression of tumors. Here, we identified for the first time that PAICS was upregulated in GC. Moreover, knockdown of PAICS in GC cells significantly inhibited cell growth in vitro and in vivo. Analysis of the detailed mechanism revealed that PAICS was involved in the DDR by interacting with HDAC1/2. In addition, the interaction between PAICS and HDAC1/2 was increased significantly in the presence of CDDP-induced DNA damage. Knockdown of PAICS impaired the deacetylase activity of HDAC1/2, reducing the expression and recruitment to DNA damage sites of DAD51, eventually interfering with DNA damage repair. More importantly, the deficiency of PAICS increased the sensitivity of GC cells to CDDP both in vitro and in vivo (Fig. 8).

Our results showed that both HR and NHEJ efficiency were inhibited with PAICS knockdown. We further explored the detailed mechanism and found that knockdown of PAICS reduced the expression of DAD51 and inhibited the recruitment of DAD51 to DNA damage sites. DAD51 is well-known as an important DNA damage repair protein in HR repair^37–39^. The deficiency of DAD51 expression or its recruitment to DNA damage sites can greatly reduce the repair efficiency of HR^37–39^. Our results suggested that PAICS regulated HR by targeting DAD51. It has been reported that the HDAC1/2, as an essential deacetylase of histone, plays an important role in promoting DNA damage and repair^40,41^. Previous studies reported that the inhibition of HDAC1/2 activity affected the recruitment of NHEJ downstream genes, such as Ku70/80 and XRCC4 to the DNA damage sites, thus impairing the repair efficiency of NHEJ^41^. We found that knockdown of PAICS partially inhibited the deacetylase activity of HDAC1/2, suggested that PAICS regulated NHEJ by impairing HDAC1/2 activity. However, further studies are needed to explore the in-depth molecular mechanisms of PAICS in regulating NHEJ pathways.

In this study, we observed no obvious changes in HDAC1/2 protein levels either in CDDP-induced DDR or its recovery. Further analysis found that knockdown of PAICS significantly decreased HDAC deacetylation activity upon CDDP treatment. Notably, our results showed that knockdown of PAICS had no obvious effect on HDAC deacetylation activity in the absence of CDDP treatment. Moreover, the interaction of PAICS and HDAC1/2 was weak in GC cells without CDDP stimulation, which was significantly enhanced in CDDP-induced DNA damage. Therefore, we speculate that PAICS might impair HDAC1/2 deacetylation activity by interacting with the functional domains of HDAC1/2, which is regulated with a “switch-like” mechanism. Under normal conditions, PAICS interacts HDAC1/2 weakly and PAICS deficiency does not impaired HDAC1/2 deacetylation activity; upon DNA damage, the interaction of PAICS and HDAC1/2 is enhanced, which induces “switch on” and enhances HDAC deacetylation activity. In this case, knockdown of PAICS causes reduced HDAC deacetylation activity and series of events. The detailed mechanism of PAICS in regulating HDAC1/2 deacetylation activity remains unclear and needs further exploration.

Cisplatin is widely used as a first-line chemotherapy drug for patients with GC. However, the clinical application of this agent is largely limited because of inevitable drug toxicity and resistance^42^. HDAC inhibitors (HDACis) singly or in combination with other chemotherapy drugs have been demonstrated to be promising therapeutic strategies for cancer^43–45^. Despite of numerous HDACis being evaluated in clinical trials, SAHA and romidepsin have been approved by the FDA for treating cutaneous T-cell lymphoma^46,47^. HDACis have also been reported to enhance the cytotoxicity of cisplatin on tumor cells^34^. Consistently, we observed that knockdown of PAICS impaired HDAC1/2 deacetylase activity, thus increasing the sensitivity of GC cells to cisplatin. Cisplatin forms a cross-linked complex with DNA, which can induce DNA damage that leads to cancer cell death and apoptosis^48^. Increased histone acetylation during DNA damage repair causes the structure of DNA to change from tight to loose, providing favorable conditions for the recruitment of DNA damage repair proteins to DNA damage sites^29^. Under PAICS-deficient conditions, DNA damage could not be repaired immediately together when HDAC1/2 deacetylase activity was also impaired, maintaining the chromatin in an open state. This loose structure of chromatin promotes the binding of cisplatin to DNA, which induces DNA damage and increases cell death. Therefore, epigenetic chromatin modification may be an important factor that increases cisplatin sensitivity caused by PAICS deficiency.

Taken together, our findings support that PAICS, a novel oncogene of GC, is involved in the DDR by interacting with HDAC1/2. In addition, PAICS deficiency enhances the sensitivity of GC cells to CDDP both in vitro and in vivo, suggesting that PAICS is a new therapeutic target for patients with GC.

## Materials and methods

### Cell culture and CDDP administration

HEK293T cells and human GC cell lines (AGS and SGC-7901) were purchased from the American Type Culture Collection (Manassas, VA, USA). The normal gastric epithelial cell line GES-1 was purchased from the Cell Bank of the Chinese Academy of Sciences (Shanghai, China). Cell lines were certificated by STR analysis (Shanghai Biotechnology Co., Ltd., Shanghai, China). AGS, SGC-7901 and GES-1 cells were cultured in RPMI 1640 medium (Gibco, CA, USA) and HEK293T cells were cultured in Dulbecco’s modified Eagle’ medium (Gibco). Both media were supplemented with 10% fetal bovine serum (Gibco) and 1% penicillin/streptomycin (Gibco). All cells were maintained in a humidified incubator at 37 °C with 5% CO_2_. Cisplatin (CDDP) was purchased from Sigma (St. Louis, MO, USA) and dissolved in saline solution at a concentration of 1 mg/ml. For induction of DNA damage, cells were treated with 10 µg/mL CDDP for 12 h followed by a 2-h recovery.

### Plasmids and transfection

Plasmids and transfection is described in detail in Supplementary Methods.

### Gene expression analysis using publicly available datasets

Gene expression level of PAICS in GC and adjacent normal samples was obtained from GEPIA (http://gepia.cancer-pku.cn/detail.php), UALCAN (http://ualcan.path.uab.edu), Kaplan–Meier plotter (http://kmplot.com/analysis/) and Human Protein Atlas (https://www.proteinatlas.org) web portal, which provide histopathological images and boxplots depicting PAICS expression based on TCGA datasets. CancerSEA (http://biocc.hrbmu.edu.cn/CancerSEA/) that depicts single-cell functional status maps was used to analyse the roles of PAICS in DNA damage and repair.

### Cell proliferation, colony formation, apoptosis, caspase-3 activity aassays, cell-cycle analysis, and flow cytometric analysis of Ki67

The assays were performed as detailed in Supplementary Methods.

### Tumor xenograft model

The male BALB/c nude mice (5 weeks) were purchased from the Beijing Vital River Laboratory Animal Technology Co. Ltd. (Beijing, China). To determine the role of PAICS in vivo, AGS cells (5 × 10^6^ cells/mouse) with stable knockdown of PAICS and the corresponding control were injected subcutaneously into the mice (7 mice/group). Tumor size was measured every seven days and calculated using the following formula: volume = (length × width^2^) × 0.5. The mice were sacrificed after 4–5 weeks and tumors were removed, weighed, fixed for immunohistochemistry detection of Ki67 (#9449, Cell Signaling Technology, CST, Danvers, MA, USA). For drug sensitivity assay, mice were injected subcutaneously with AGS cells (5 × 10^6^ cells/mouse). When the tumors were measurable, the mice (4 mice/group) were randomly and blindly divided into the control group receiving saline solution (100 µL) and CDDP-treated group receiving CDDP (5 mg/kg dissolved in 100 µL saline solution) by intraperitoneal injection three times a week for 2–3 weeks. All animal studies were approved by the Animal Research Ethics Committee of Shanghai Tenth People’s Hospital (SHHDSRMYY-2019-4642).

### RNA isolation, qRT-PCR, western blot analysis, and histone deacetylase activity assay

The assays were performed as detailed in Supplementary Methods.

### HR and NHEJ assays

HEK293T cells were co-transfected with siPAICS or siCON (NC), together with DR-GFP (the negative control), GFP (the positive control) or DR-GFP + Isce-I (HR) or NHEJ, separately. After 48 h of transfection, cells were collected and subjected to examine the percentage of GFP (GFP%) using the FACSC anto^TM^ II flow cytometer (BD). The HR or NHEJ repair efficiency was calculated using the following formula: (GFP% of HR/NHEJ − GFP% of negative control)/GFP% of positive control.

### Immunofluorescence assay

Cells cultured on confocal dishes were fixed with 4% paraformaldehyde (Sigma) for 15 min at room temperature, and permeabilized with 0.5% Triton X-100 for 20 min at 37 °C. After blocking with 5% BSA for 1 h at room temperature, cells were incubated with the primary antibodies against PAICS (Abclone, 1:100), γH2AX (CST, 1:200) and DAD51 (Abcam, 1:100) at 4 °C overnight. The next day, cells were incubated with corresponding secondary antibodies against Cy™3 AffiniPure Donkey Anti-Mouse IgG (H+L) or Alexa Fluor 647 AffiniPure Goat Anti-Rabbit IgG (H+L) (Jackson) at 37 °C for 30 min. 4′,6-diamidino-2-phenylindole (DAPI, Sigma) was then applied to stain the nuclei. Finally, the images of immunofluorescence were obtained with the confocal laser-scanning microscope (Carl Zeiss, Jena, Germany).

### Neutral comet assay

AGS and SGC-7901 cells with stable knockdown of PAICS and the corresponding control were collected and mixed with 0.5% low-melting point agarose, followed by being added to the slides covered with 1% normal-melting agarose. After solidifying at 4 °C for 20 min, the slides were immersed in the iced neutral lysis buffer at 4 °C for 4 h. Then, the electrophoresis was performed with untwisted DNA in the pre-cooled neutral electrophoresis buffer. Finally, the slides were stained with SYBR Green I and photographed by the inverted fluorescence microscope (Carl Zeiss). DNA damage was visualized as the percentage of tail DNA and tail moment.

### Immunoprecipitation

Cells were collected and lysed with the NETN buffer (20 mM Tris-HCl pH 8.0, 0.5% Nonidet P-40, 200 mM NaCl, 1 mM EDTA) containing protease and phosphatase inhibitors (Sigma) for 30 min on ice. Then, equivalent proteins (1000 μg) were incubated with specific antibodies together with 30 μL protein A/G plus-agarose (Santa Cruz, Texas, USA) rocking at 4 °C overnight. The next day, the immunocomplexes were washed five times with cold NETN buffer, boiled in sample buffer and finally subjected to western blot analysis.

### Statistical analysis

All data are shown as mean ± standard deviation (SD) from three independent experiments. Statistical analysis was performed using SPSS v22.0 (IBM, Armonk, NY, USA). Differences were analyzed by Student’s *t*-test for two groups or one-way ANOVA for multiple groups. All experiments were performed three times and the *p* values < 0.05 were considered statistically significant.

## Supplementary information

Supplementary Fig. 1

Supplementary Fig. 2

Supplementary Fig. 3

Supplementary Fig. 4

Supplementary Table S1

Supplementary Figure And Table Legends

Supplementary Methods

## Data Availability

The data that support the findings of this study are available from the corresponding author upon reasonable request.

## References

[1] Bray, F. et al. Global cancer statistics 2018: GLOBOCAN estimates of incidence and mortality worldwide for 36 cancers in 185 countries. *CA Cancer J. Clin.* **68**, 394–424 (2018). 30207593 10.3322/caac.21492

[2] Smyth, E. C. et al. Gastric cancer: ESMO Clinical Practice Guidelines for diagnosis, treatment and follow-up. *Ann. Oncol.* **27**, v38–v49 (2016). 27664260 10.1093/annonc/mdw350

[3] Haj Mohammad, N. et al. Optimal first-line chemotherapeutic treatment in patients with locally advanced or metastatic esophagogastric carcinoma: triplet versus doublet chemotherapy: a systematic literature review and meta-analysis. *Cancer Metastasis Rev.* **34**, 429–441 (2015). 26267802 10.1007/s10555-015-9576-y PMC4573655

[4] Lawrence, M. S. et al. Mutational heterogeneity in cancer and the search for new cancer-associated genes. *Nature* **499**, 214–218 (2013). 23770567 10.1038/nature12213 PMC3919509

[5] Li, S. X. et al. Octameric structure of the human bifunctional enzyme PAICS in purine biosynthesis. *J. Mol. Biol.* **366**, 1603–1614 (2007). 17224163 10.1016/j.jmb.2006.12.027

[6] Zhou, S. et al. Roles of highly expressed PAICS in lung adenocarcinoma. *Gene* **692**, 1–8 (2019). 30641222 10.1016/j.gene.2018.12.064

[7] Meng, M., Chen, Y., Jia, J., Li, L. & Yang, S. Knockdown of PAICS inhibits malignant proliferation of human breast cancer cell lines. *Biol. Res.* **51**, 24 (2018). 30097015 10.1186/s40659-018-0172-9 PMC6086025

[8] Chakravarthi, B. et al. Expression and role of PAICS, a de novo purine biosynthetic gene in prostate cancer. *Prostate* **78**, 693–694 (2018). 29744932 10.1002/pros.23533

[9] Chakravarthi, B. et al. A role for de novo purine metabolic enzyme PAICS in bladder cancer progression. *Neoplasia* **20**, 894–904 (2018). 30121007 10.1016/j.neo.2018.07.006 PMC6098199

[10] Goswami, M. T. et al. Role and regulation of coordinately expressed de novo purine biosynthetic enzymes PPAT and PAICS in lung cancer. *Oncotarget* **6**, 23445–23461 (2015). 26140362 10.18632/oncotarget.4352 PMC4695129

[11] Zaza, G. et al. Acute lymphoblastic leukemia with TEL-AML1 fusion has lower expression of genes involved in purine metabolism and lower de novo purine synthesis. *Blood* **104**, 1435–1441 (2004). 15142881 10.1182/blood-2003-12-4306

[12] Huang, Q., Liu, F. & Shen, J. Bioinformatic validation identifies candidate key genes in diffuse large-B cell lymphoma. *Per Med.* **16**, 313–323 (2019). 31331250 10.2217/pme-2018-0068

[13] Spampinato, C. P. Protecting DNA from errors and damage: an overview of DNA repair mechanisms in plants compared to mammals. *Cell Mol. Life Sci.* **74**, 1693–1709 (2017). 27999897 10.1007/s00018-016-2436-2 PMC11107726

[14] Sugasawa, K. Molecular mechanisms of DNA damage recognition for mammalian nucleotide excision repair. *DNA Repair* **44**, 110–117 (2016). 27264556 10.1016/j.dnarep.2016.05.015

[15] Jackson, S. P. & Bartek, J. The DNA-damage response in human biology and disease. *Nature* **461**, 1071–1078 (2009). 19847258 10.1038/nature08467 PMC2906700

[16] Chatterjee, N. & Walker, G. C. Mechanisms of DNA damage, repair, and mutagenesis. *Environ. Mol. Mutagen* **58**, 235–263 (2017). 28485537 10.1002/em.22087 PMC5474181

[17] Wilson, M. D. & Durocher, D. Reading chromatin signatures after DNA double-strand breaks. *Philos. Trans. R Soc. Lond. B Biol. Sci.* **372**, 20160280 (2017). 10.1098/rstb.2016.0280 PMC5577458 28847817

[18] Stucki, M. et al. MDC1 directly binds phosphorylated histone H2AX to regulate cellular responses to DNA double-strand breaks. *Cell* **123**, 1213–1226 (2005). 16377563 10.1016/j.cell.2005.09.038

[19] Shiloh, Y. & Ziv, Y. The ATM protein kinase: regulating the cellular response to genotoxic stress, and more. *Nat. Rev. Mol. Cell Biol.* **14**, 197–210 (2013). 23486281 10.1038/nrm3546

[20] Matsuoka, S. et al. ATM and ATR substrate analysis reveals extensive protein networks responsive to DNA damage. *Science* **316**, 1160–1166 (2007). 17525332 10.1126/science.1140321

[21] Stucki, M. & Jackson, S. P. gammaH2AX and MDC1: anchoring the DNA-damage-response machinery to broken chromosomes. *DNA Repair* **5**, 534–543 (2006). 16531125 10.1016/j.dnarep.2006.01.012

[22] Gottlieb, T. M. & Jackson, S. P. The DNA-dependent protein kinase: requirement for DNA ends and association with Ku antigen. *Cell* **72**, 131–142 (1993). 8422676 10.1016/0092-8674(93)90057-w

[23] Menon, V. & Povirk, L. F. End-processing nucleases and phosphodiesterases: An elite supporting cast for the non-homologous end joining pathway of DNA double-strand break repair. *DNA Repair* **43**, 57–68 (2016). 27262532 10.1016/j.dnarep.2016.05.011

[24] Wright, W. D., Shah, S. S. & Heyer, W. D. Homologous recombination and the repair of DNA double-strand breaks. *J. Biol. Chem.* **293**, 10524–10535 (2018). 29599286 10.1074/jbc.TM118.000372 PMC6036207

[25] Truong, L. N. et al. Homologous recombination is a primary pathway to repair DNA double-strand breaks generated during DNA rereplication. *J. Biol. Chem.* **289**, 28910–28923 (2014). 25160628 10.1074/jbc.M114.576488 PMC4200250

[26] McGlynn, P. & Lloyd, R. G. Recombinational repair and restart of damaged replication forks. *Nat. Rev. Mol. Cell Biol.* **3**, 859–870 (2002). 12415303 10.1038/nrm951

[27] Roos, W. P., Thomas, A. D. & Kaina, B. DNA damage and the balance between survival and death in cancer biology. *Nat. Rev. Cancer* **16**, 20–33 (2016). 26678314 10.1038/nrc.2015.2

[28] Tjeertes, J. V., Miller, K. M. & Jackson, S. P. Screen for DNA-damage-responsive histone modifications identifies H3K9Ac and H3K56Ac in human cells. *EMBO J.* **28**, 1878–1889 (2009). 19407812 10.1038/emboj.2009.119 PMC2684025

[29] Van, H. T. & Santos, M. A. Histone modifications and the DNA double-strand break response. *Cell Cycle* **17**, 2399–2410 (2018). 30394812 10.1080/15384101.2018.1542899 PMC6342081

[30] Adimoolam, S. et al. HDAC inhibitor PCI-24781 decreases DAD51 expression and inhibits homologous recombination. *Proc. Natl Acad. Sci. USA* **104**, 19482–19487 (2007). 18042714 10.1073/pnas.0707828104 PMC2148315

[31] Krumm, A. et al. Enhanced histone deacetylase activity in malignant melanoma provokes DAD51 and FANCD2-triggered drug resistance. *Cancer Res.* **76**, 3067–3077 (2016). 26980768 10.1158/0008-5472.CAN-15-2680

[32] Lai, T. H. et al. HDAC inhibition induces microRNA-182, which targets DAD51 and impairs HR repair to sensitize cells to sapacitabine in acute myelogenous leukemia. *Clin. Cancer Res.* **22**, 3537–3549 (2016). 26858310 10.1158/1078-0432.CCR-15-1063 PMC4947457

[33] Zhao, J. et al. Histone deacetylases 1 and 2 cooperate in regulating BRCA1, CHK1, and DAD51 expression in acute myeloid leukemia cells. *Oncotarget* **8**, 6319–6329 (2017). 28030834 10.18632/oncotarget.14062 PMC5351634

[34] Diyabalanage, H. V., Granda, M. L. & Hooker, J. M. Combination therapy: histone deacetylase inhibitors and platinum-based chemotherapeutics for cancer. *Cancer Lett.* **329**, 1–8 (2013). 23032720 10.1016/j.canlet.2012.09.018 PMC3546543

[35] Liu, X. et al. HDAC1 silencing in ovarian cancer enhances the chemotherapy response. *Cell Physiol. Biochem* **48**, 1505–1518 (2018). 30071534 10.1159/000492260

[36] Pchejetski, D. et al. Histone deacetylases as new therapy targets for platinum-resistant epithelial ovarian cancer. *J. Cancer Res Clin. Oncol.* **142**, 1659–1671 (2016). 26560874 10.1007/s00432-015-2064-5 PMC4954831

[37] Godin, S. K., Sullivan, M. R. & Bernstein, K. A. Novel insights into DAD51 activity and regulation during homologous recombination and DNA replication. *Biochem Cell Biol.* **94**, 407–418 (2016). 27224545 10.1139/bcb-2016-0012 PMC5045787

[38] Holthausen, J. T., Wyman, C. & Kanaar, R. Regulation of DNA strand exchange in homologous recombination. *DNA Repair* **9**, 1264–1272 (2010). 20971042 10.1016/j.dnarep.2010.09.014

[39] Krejci, L., Altmannova, V., Spirek, M. & Zhao, X. Homologous recombination and its regulation. *Nucleic Acids Res.* **40**, 5795–5818 (2012). 22467216 10.1093/nar/gks270 PMC3401455

[40] Murr, R. et al. Histone acetylation by Trrap-Tip60 modulates loading of repair proteins and repair of DNA double-strand breaks. *Nat. Cell Biol.* **8**, 91–99 (2006). 16341205 10.1038/ncb1343

[41] Miller, K. M. et al. Human HDAC1 and HDAC2 function in the DNA-damage response to promote DNA nonhomologous end-joining. *Nat. Struct. Mol. Biol.* **17**, 1144–1151 (2010). 20802485 10.1038/nsmb.1899 PMC3018776

[42] Venerito, M., Vasapolli, R., Rokkas, T. & Malfertheiner, P. Gastric cancer: epidemiology, prevention, and therapy. *Helicobacter* **23**(Suppl 1), e12518 (2018). 30203589 10.1111/hel.12518

[43] Laschanzky, R. S. et al. Selective inhibition of histone deacetylases 1/2/6 in combination with gemcitabine: a promising combination for pancreatic cancer therapy. *Cancers* **11**, 1327 (2019). 10.3390/cancers11091327 PMC6770665 31500290

[44] Schizas, D. et al. Concept of histone deacetylases in cancer: Reflections on esophageal carcinogenesis and treatment. *World J. Gastroenterol.* **24**, 4635–4642 (2018). 30416311 10.3748/wjg.v24.i41.4635 PMC6224471

[45] Zhang, H., Shang, Y. P., Chen, H. Y. & Li, J. Histone deacetylases function as novel potential therapeutic targets for cancer. *Hepatol. Res.* **47**, 149–159 (2017). 27457249 10.1111/hepr.12757

[46] Furumai, R. et al. FK228 (depsipeptide) as a natural prodrug that inhibits class I histone deacetylases. *Cancer Res.* **62**, 4916–4921 (2002). 12208741

[47] Grant, C. et al. Romidepsin: a new therapy for cutaneous T-cell lymphoma and a potential therapy for solid tumors. *Expert Rev. Anticancer Ther.* **10**, 997–1008 (2010). 20645688 10.1586/era.10.88 PMC6361116

[48] Nikolova, T., Kiweler, N. & Kramer, O. H. Interstrand crosslink repair as a target for HDAC inhibition. *Trends Pharm. Sci.* **38**, 822–836 (2017). 28687272 10.1016/j.tips.2017.05.009

