# Supplementary figures and images for: PAICS contributes to gastric carcinogenesis and participates in DNA damage response by interacting with histone deacetylase 1/2
Source: Cell Death Dis. 2020 Jul 6;11(7):507. doi: 10.1038/s41419-020-2708-5 (PMC7338359; doi:10.1038/s41419-020-2708-5)

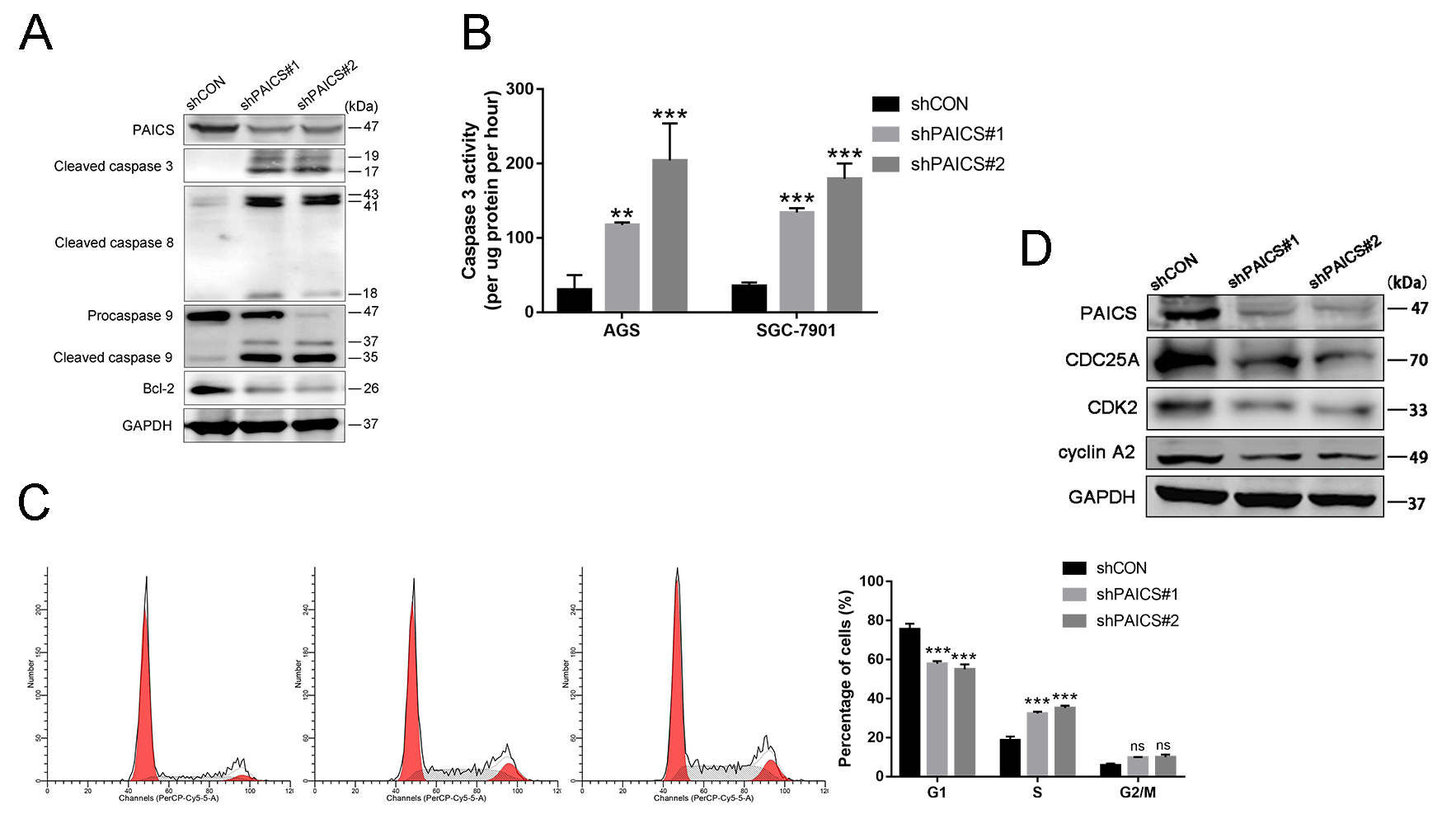

Supplement: Supplementary file 1 — Supplementary Fig. 1 [file 41419_2020_2708_MOESM1_ESM.tif]

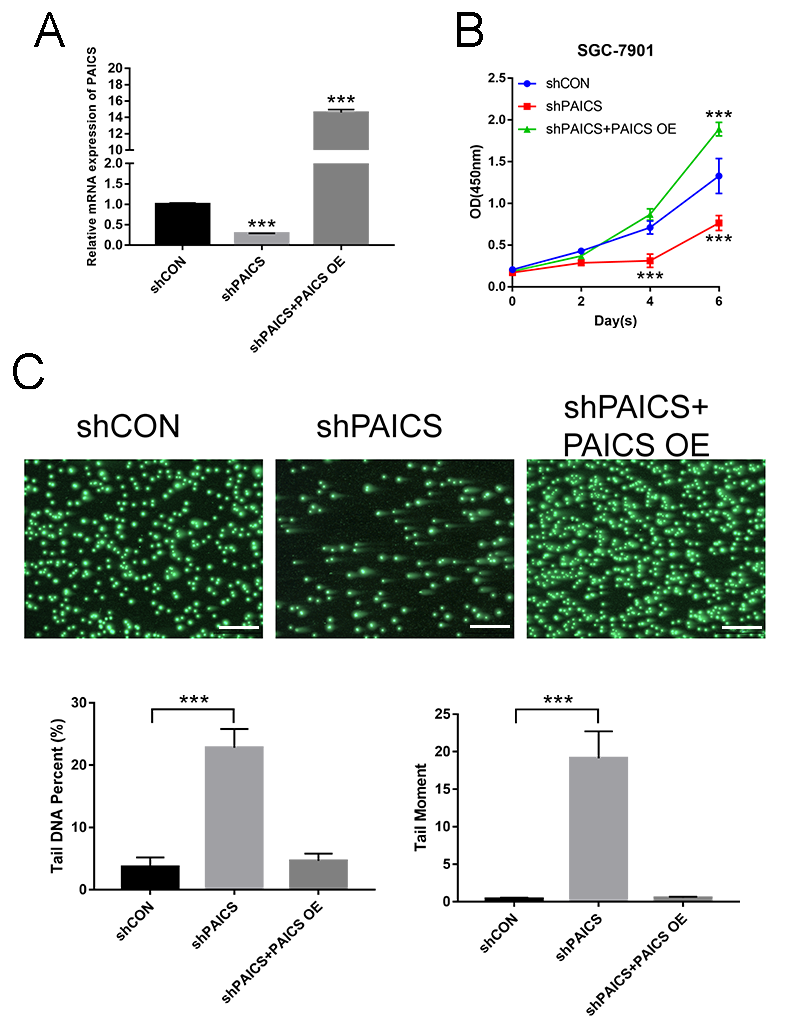

Supplement: Supplementary file 2 — Supplementary Fig. 2 [file 41419_2020_2708_MOESM2_ESM.tif]

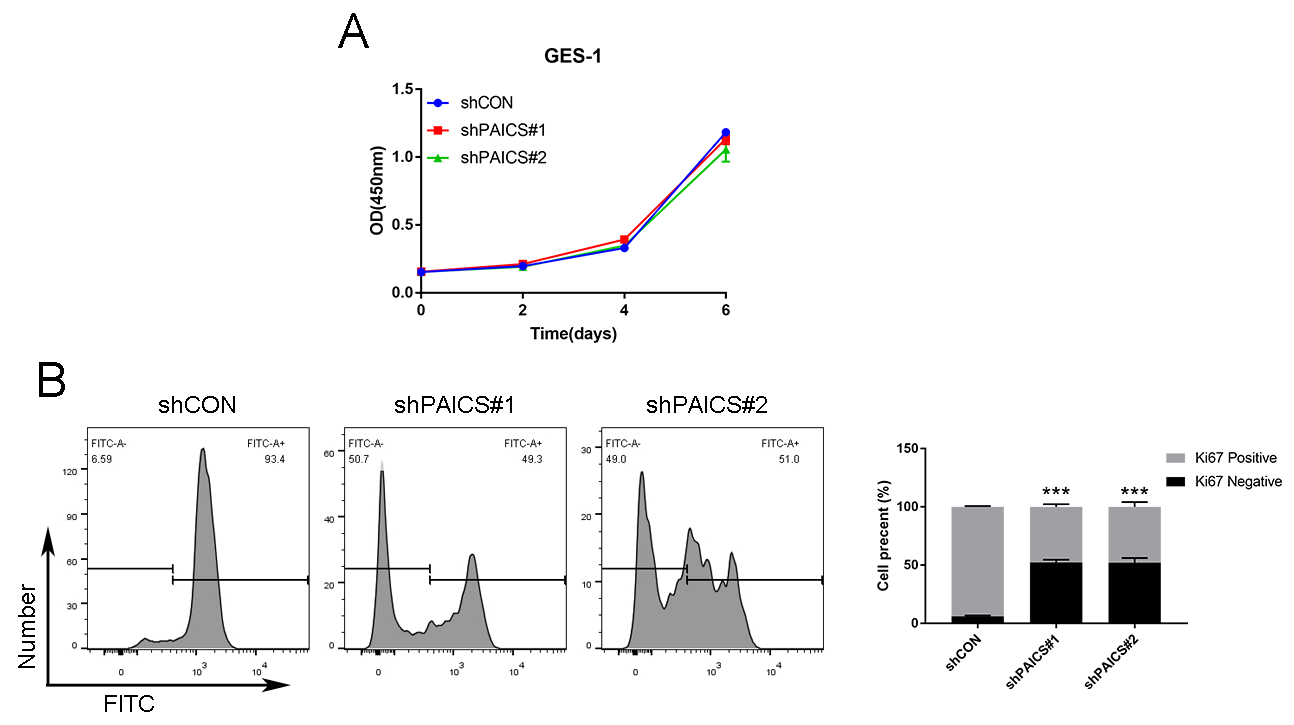

Supplement: Supplementary file 3 — Supplementary Fig. 3 [file 41419_2020_2708_MOESM3_ESM.tif]

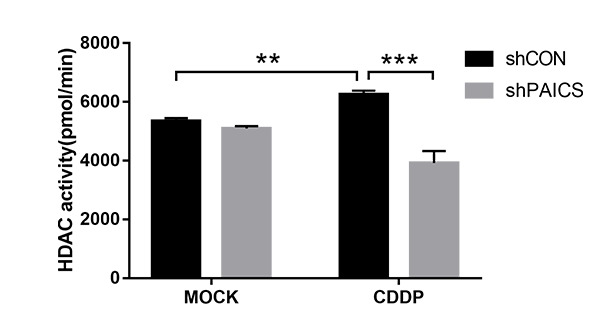

Supplement: Supplementary file 4 — Supplementary Fig. 4 [file 41419_2020_2708_MOESM4_ESM.tif]
