# Supplementary material for: PAICS contributes to gastric carcinogenesis and participates in DNA damage response by interacting with histone deacetylase 1/2
Source: Cell Death Dis. 2020 Jul 6;11(7):507. doi: 10.1038/s41419-020-2708-5 (PMC7338359; doi:10.1038/s41419-020-2708-5)
Supplement: Supplementary file 6 — Supplementary Figure And Table Legends [file 41419_2020_2708_MOESM6_ESM.docx]

**Supplementary Table S1. List of PAICS interacting proteins identified by LC-MS/MS mass spectrometry.**

**Supplementary Figure Legends**

**Supplementary Fig. 1 PAICS knockdown induces apoptosis and cell-cycle arrest of GC cells.**

**a** Western blot analysis of the related apoptotic proteins, including cleaved caspases 3/8/9 and Bcl 2 in SGC-7901 cells with stable knockdown of PAICS (shPAICS#1, shPAICS#2) and the corresponding control (shCON) cells. **b** The activity assay on Caspase 3 in shPAICS#1-, shPAICS#2- and shCON-SGC-7901 cells. **c** Flow cytometric analysis of cell cycle distribution in shPAICS#1-, shPAICS#2- and shCON-SGC-7901 cells. Columns (right panel) present percentage of cells arrested in G1, S and G2/M phase of the cell cycle. **d** Western blot analysis of the S phase-related proteins, including CDC25A, cyclin A2 and CDK2, in shPAICS#1-, shPAICS#2- and shCON-SGC-7901 cells. Data represent the mean ± SD from three independent experiments, ****p*< 0.001, ***p*< 0.01. NS, no significance.

**Supplementary Fig. 2** **PAICS overexpression rescues the growth-inhibitory effect and DNA damage of GC cells induced by PAICS knockdown.**

**a** qRT-PCR confirming the efficiency of PAICS knockdown and overexpression (OE) in SGC-7901 cells. **b,c** Rescue assays confirming the effects of PAICS on cell proliferation **b** and DNA damage **c** in SGC-7901 cells. Data represent the mean ± SD from three independent experiments, ****p*< 0.001.

**Supplementary Fig. 3 PAICS knockdown inhibits GC cell proliferation but has little effect on normal gastric cells.**

**a** CCK8 showing cell proliferation in shCON- or shPAICS- normal gastric cells GES-1. **b** Flow cytometric analysis of Ki67 in shCON- or shPAICS-SGC-7901 cells. Data represent the mean ± SD from three independent experiments, ****p*< 0.001.

**Supplementary Fig. 4 PAICS knockdown decreases HDAC deacetylation activity upon CDDP treatment.**

HDAC deacetylation activity of shCON- or shPAICS-SGC-7901 cells stimulated with or without CDDP (10 µg/ mL for 12 h followed by a 2 h recovery). Data represent the mean ± SD from three independent experiments, ****p*< 0.001, ***p*< 0.01.
