# Supplementary material for: PAICS contributes to gastric carcinogenesis and participates in DNA damage response by interacting with histone deacetylase 1/2
Source: Cell Death Dis. 2020 Jul 6;11(7):507. doi: 10.1038/s41419-020-2708-5 (PMC7338359; doi:10.1038/s41419-020-2708-5)
Supplement: Supplementary file 7 — Supplementary Methods [file 41419_2020_2708_MOESM7_ESM.docx]

**Plasmids and transfection**

Lentivirus constructing of PAICS knockdown or the scramble control (shPAICS-1 or shPAICS-2, shCON) were obtained from GenePharma (Suzhou, China). GC cells were plated in six-well plates at 50% confluence and infected with PAICS-knockdown or a scramble control lentivirus for 24 h. Pools of stable transductions were generated by selection using 2 μg/ml puromycin (Sigma) for 2 weeks. Plasmids for GFP tagged PAICS, GFP-vector, Flag tagged HDAC1 and corresponding Flag-vector were obtained from GenePharma. Small interfering RNAs (siRNAs) against PAICS and the negative control were synthesized by GenePharma. Transient transfections of plasmids to HEK293T for immunoprecipitation or to PAICS-knockdown and the control GC cells for rescue assays were performed using the Lipofectamine 2000 kit (Invitrogen, Carlsbad, USA) according to the manufacturer’s instructions.

**RNA isolation and qRT-PCR**

Total RNA was extracted from cultured cell lines by Trizol reagent (Invitrogen) and used to synthesize cDNA with the PrimeScript^TM^ RT reagent Kit (TaKaRa, Dalian, China) according to the manufacturer’s instructions. qRT-PCR was performed on an ABI 7900HT PCR sequencer (Applied Biosystems, Massachusetts, USA) using TB Green^®^ Premix Ex Taq™ II (TaKaRa). Primers sequences used for qRT-PCR were as follows: PAICS, Forward: 5′- GTGGCAGGCAGAAGTAATGG -3′. Reverse: 5′- CACATCCTGAACTCCCCAGT- 3′. GADPH, Forward: 5′- TGCACCACCAACTGCTTAGC -3′. Reverse: 5′- GGCATGGACTGTGGTCATGAG -3′. Rad51, Forward: 5′- ACGGAGTCTCGCTCTGTTGC -3′. Reverse: 5′- GGAATTCTCCAAACATTTCT -3′. Fold changes in mRNA expression were calculated using 2^-ΔΔCt^ and standardized based on GAPDH.

**Western blot analysis**

Cells were collected and lysed with RIPA buffer containing protease and phosphatase inhibitors (Sigma) to extract proteins. Equivalent proteins were separated using proper SDS-PAGE gel and transferred to nitrocellulose membranes. For western blot analysis, the membranes were blocked with 5% bovine serum albumin (BSA) for 1 h at room temperature, followed by incubated with primary antibodies against PAICS (A6450, Abclone, Wuhan, China), GAPDH (ab128915, Abcam, Cambridge, MA, USA), cleaved caspase-3 (#9661s, Cell Signaling Technology, CST, Danvers, MA, USA), cleaved caspase-8 (#9496s, CST), caspase-9 (ab202068, Abcam), Bcl-2 (ab32124, Abcam), CDC25A (#3652S, CST), cyclin A2 (ab137769, Abcam), CDK2 (#2546S, CST), rH2AX (ab2893, Abcam), Rad51 (ab133534, Abcam), HDAC1 (ab31236, Abcam), HDAC2 (ab32117, Abcam), GFP (ab290, Abcam), FLAG (#8146S, CST), Histone H3 (ab176842, Abcam) and Histone H3(acetyl K56, ab241461, Abcam) at 4 °C overnight. The next day, the membranes were incubated with fluorescence-conjugated secondary antibodies. Finally, the signals were detected using the Odyssey two-color infrared fluorescence imaging system (LI-COR, Lincoln, USA).

**Cell proliferation assay**

Cell proliferation was assessed using the Cell Counting Kit-8 (CCK8, Beyotime, Haimen, China). Pretreated cells (1 × 10^3^ cells/well) were seeded into a 96-well plate and severally cultured for 1, 2, 3, 4, 5 and 6 days. In drug sensitivity experiments, CDDP (Sigma) was added into the wells at the indicated concentration after cell attachment for 12 h. The absorbance was measured at 450 nm using a multi-function microplate reader (BioTek, USA) after cells were incubated with CCK8 at 37 °C for 2 h.

**Colony formation assay**

Pretreated cells (500 cells/well) were seeded into 12-well plates. After incubation for 14 days, the colonies were fixed in absolute ethanol for 15 minutes, stained with 0.1% crystal violet solution for 30 min, and photographed with a digital camera. Finally, the colony numbers were counted by the Image J software (National Institutes of Health, Bethesda, MD, USA).

**Flow cytometric analysis of apoptosis**

The quantification of apoptotic cells was determined by flow cytometry using an AnnexinV-APC / 7-AAD double staining Kit (BD Biosciences, Franklin Lakes, NJ, USA) according to the manufacturer’s instructions. Briefly, cells were collected and washed with ice-cold phosphate-buffered saline (PBS). Then, Cells were stained with Annexin V-APC and 7-AAD in the dark at room temperature for 20 min. Finally, cell apoptosis was detected using the FACSCanto^TM^ II flow cytometer (BD).

**Caspase-3 activity assay**

SGC-7901 cells with stable knockdown of PAICS and the corresponding control were harvested and washed with ice-cold PBS. Then, the activity assay on Caspase 3 was detected using a Caspase 3 Activity Assay Kit (Beyotime), according to the manufacturer’s instructions. Finally, the absorbance was measured at 405 nm using a multi-function microplate reader (BioTek).

**Cell-cycle analysis**

SGC-7901 cells with stable knockdown of PAICS and the corresponding control were synchronized, collected and fixed with 75% ethanol at −20 °C overnight. Then, cells were washed with ice-cold PBS and stained with PI/RNase staining buffer (BD Biosciences) in the dark at room temperature for 15 min. Finally, the stained cells were detected with the FACSCanto^TM^ II flow cytometer (BD), and the percentage of cell cycle distribution was analyzed using ModFit LT software (Verity Software House, Topsham, ME, USA).

**Flow cytometric analysis of Ki67**

SGC-7901 cells with stable knockdown of PAICS and the corresponding control were harvested and washed with ice-cold PBS. Then, cells were stained with primary labelled Ki67 antibody (ab197234, Abcam) at room temperature for 30 min. Finally, the stained cells were detected with the FACSCanto^TM^ II flow cytometer (BD), and the percentage of Ki67 positive or negative cells was analyzed using FlowJo software (Tree Star, Inc., Ashland, OR, USA).

**Histone deacetylase activity assay**

SGC-7901 cells with stable knockdown of PAICS and the corresponding control seeded into 96-well plates were stimulated with or without CDDP (10 µg/ mL for 12 h followed by a 2 h recovery). Then, the histone deacetylases deacetylation activity was detected using the HDAC assay kit (Active Motif, Shanghai, China), according to the manufacturer’s instructions. Finally, the absorbance was measured at 405 nm using a multi-function microplate reader (BioTek).
